# Integration of pan-cancer transcriptomics with RPPA proteomics reveals mechanisms of epithelial-mesenchymal transition

**DOI:** 10.1371/journal.pcbi.1005911

**Published:** 2018-01-02

**Authors:** Simon Koplev, Katie Lin, Anders B. Dohlman, Avi Ma’ayan

**Affiliations:** Department of Pharmacological Sciences, Mount Sinai Center for Bioinformatics, Icahn School of Medicine at Mount Sinai, One Gustave L. Levy Place, New York, NY, United States of America; University Hospital RWTH Aachen, GERMANY

## Abstract

Integrating data from multiple regulatory layers across cancer types could elucidate additional mechanisms of oncogenesis. Using antibody-based protein profiling of 736 cancer cell lines, along with matching transcriptomic data, we show that pan-cancer bimodality in the amounts of mRNA, protein, and protein phosphorylation reveals mechanisms related to the epithelial-mesenchymal transition (EMT). Based on the bimodal expression of E-cadherin, we define an EMT signature consisting of 239 genes, many of which were not previously associated with EMT. By querying gene expression signatures collected from cancer cell lines after small-molecule perturbations, we identify enrichment for histone deacetylase (HDAC) inhibitors as inducers of EMT, and kinase inhibitors as mesenchymal-to-epithelial transition (MET) promoters. Causal modeling of protein-based signaling identifies putative drivers of EMT. In conclusion, integrative analysis of pan-cancer proteomic and transcriptomic data reveals key regulatory mechanisms of oncogenic transformation.

## Author summary

Profiling molecular and phenotypic characteristics of large collections of cancer cell lines can be used to identify distinct and common oncogenic pathways across cancer types. So far, most large-scale data obtained from cancer cell lines have been at the genomic, transcriptomic, and phenotypic levels. Recently, high-quality data at the level of cell signaling through protein abundances and phosphorylation sites has become available. By integrating this newly generated protein data with prior transcriptomic data, and by visualizing all cancer cell lines using dimensionality reduction techniques, pan-cancer cell lines are strikingly shown to organize into a gradient of epithelial to mesenchymal types. Interestingly, many of the measured proteins and transcripts display bimodality; the expression of genes, proteins, and protein phosphorylations is either high or low, strongly suggesting that they act as molecular switches. Focusing on further characterizing molecular switches of epithelial-mesenchymal transitions, we identify candidate regulators and small molecules that can induce or reverse such transition, as well as potential causal relationships between proteins. Since the mesenchymal state of tumors is known to be associated with metastasis and later-stage cancer development, better understanding the regulatory mechanisms of epithelial-to-mesenchymal transition can lead to improved targeted therapeutics.

## Introduction

Central to the understanding of cancer cells are their epithelial or mesenchymal traits, which are governed by epithelial-mesenchymal transition (EMT). Cells that have undergone EMT display increased invasiveness and metastatic potential [1]. The transition is reversible, in that cells can also undergo mesenchymal-to-epithelial transition (MET) [2]. This plasticity plays a role in cancer progression and metastasis by increasing the capacity of cancer cells to invade and colonize at remote tissue [3]. EMT is thought to be governed by a few master regulators that induce epigenetic and transcriptional reprogramming, affecting the expression of multiple downstream genes [4]. The transition is characterized by the down-regulation of E-cadherin, which has been the gene most extensively studied, resulting in disruption of adherens junctions [5]. The inhibition of E-cadherin expression is known to be mediated by the transcription factor Snail [6]. At the *CDH1* loci, Snail recruits protein complexes containing histone deacetylases (HDACs) that deacetylate H3 and H4 histones, silencing the transcription of E-cadherin [7]. Other key transcription factors implicated in EMT are ZEB1/2 and TWIST [8]. The regulation of EMT-TFs by miR200 and miR34 constitutes a double-negative feedback mechanism [2], predicting a bistable system with binary transition between cellular states. Essentially, EMT is controlled by multiple interconnected regulatory networks, which include transcriptional and post-transcriptional mechanisms. Due to high regulatory complexity, proteomic and transcriptomic technologies provide an opportunity to obtain a more global understanding of EMT and MET, while possibly discovering additional molecular mechanisms with implications for targeted cancer therapeutics.

The reverse phase protein array (RPPA) is a high-throughput proteomics method that utilizes antibody binding to quantify protein expression and post-translational modifications including phosphorylation, acetylation, and protein cleavage. Compared to mass spectrometry proteomics, RPPA has higher sensitivity for low-abundance proteins and is characterized by increased throughput; however, it relies on high-quality antibodies, so it cannot identify proteins or post-translational modifications *de novo* [9]. Using RPPA, 736 cancer cell lines have been assayed for 450 proteins and phosphoproteins covering well-established cancer-related signaling pathways [10]. This data complements prior efforts to characterize basal mRNA expression across many of the same cancer cell lines for different cancer types [11]. In addition, tumor samples have been characterized by similar RPPA experiments for samples from the Cancer Genome Atlas (TCGA) [12], which are publicly available through the Cancer Proteomics Atlas (TCPA) [13].

Most genome-wide studies of EMT in cancer cell lines and tumors have focused on particular cancer types. Combining EMT signatures based on cell lines and tumors of multiple cancer types can identify general transcriptomic features of EMT in cancer cells, which are clinically relevant for multiple types of cancer. More recently, transcriptomic data from TCGA and Cancer Cell Line Encyclopedia (CCLE) have been used to define a pan-cancer EMT signature based on the expression of E-cadherin and Vimentin alone [14]. In this study, we integrate transcriptomics and RPPA data from multiple cancer cell lines to study pan-cancer cellular states associated with EMT.

## Results

### Transcript and protein signatures of pan-cancer cell lines organize by E-cadherin expression

**Fig 1:**
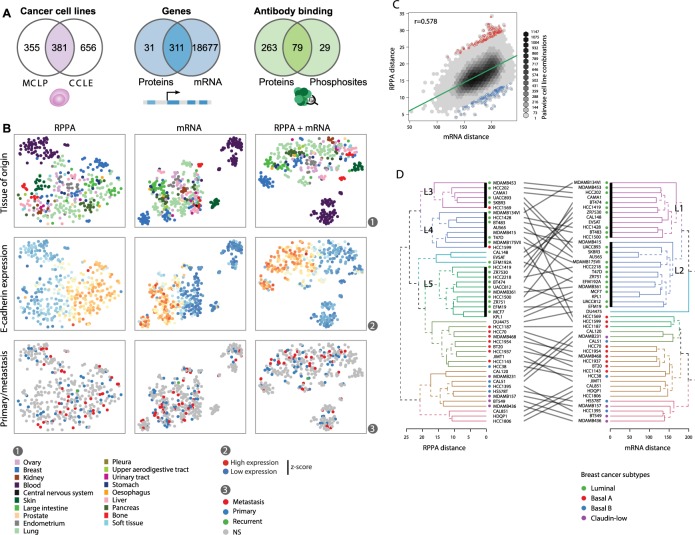
Pan-cancer cell line data from CCLE transcriptomic and reverse phase protein arrays (RPPA) cluster by tissue of origin and E-cadherin expression but not by prior metastasis classification. (**A**) Overlap of available RPPA and CCLE data with regard to cancer cell lines (left), measured transcripts and proteins (middle), and proteins measured for both basal expression and phosphorylation levels (right). The colored areas indicate data used to calculate and compare Euclidean distances between cell lines. (**B**) t-SNE plots of overlapping cancer cell lines based on protein, transcript, and equally weighted combined data. Each point represents a cell line and is colored by the tissue of origin (top), E-cadherin expression (middle), or tumor classification (bottom). NS: not specified. (**C**) Comparing pairwise distances between all cell lines using a linear model at the mRNA or protein levels. The red points show the top-100 highest residuals of cell line pairs, and the blue points the top-100 lowest residuals. (**D**) Dendrograms of breast cancer cell lines mapped for transcriptomic and RPPA data. The leaves of the trees were arranged to minimize the number of crossing lines between leaves of the two trees. L1-5 represents clusters found within the luminal subtype of breast cancer cell lines.

The Cancer Cell Line Encyclopedia (CCLE) contains 1037 cancer cell lines with profiled transcriptomes [11], and the MD Andersen Cell Line Project (MCLP) contains 736 cancer cell lines profiled by RPPA [10]. Out of these cancer cell lines, 381 have both available RPPA and microarray data. RPPA measurements are available for 450 proteins and phospho-proteins, of which 311 genes can be matched to mRNAs measured in CCLE. In the RPPA data, 79 proteins are measured both at the basal expression and phosphorylation levels (Fig 1A). To our knowledge, this data set, although far from genome-wide at the protein level, represents the largest collection of cancer cell line data measured at the transcriptional, translational, and post-translational levels.

Transcriptional profiling of human tumor samples accurately predicts the tissue of origin for common cancer types [15]. This suggests that despite oncogenic transformation, cancer cells retain cellular identity and molecular features of their ancestral cell lineage, which is a key confounding factor in pan-cancer analyses. To assess how cancer cell lines relate based on transcript and protein expression, we visualized distances between cell lines using the t-Distributed Stochastic Neighbor Embedding (t-SNE) method [16]. As expected, the cell lines are clustered predominantly by their tissue of origin for both RPPA and transcriptomic data (Fig 1B, top). Cancer types with ill-defined or multiple clusters included breast and ovary as well as cell lines from the most common targets of metastasis: liver, lung, and bone. Nonetheless, most cell lines were correctly classified by nearest neighbor classification (S1 Fig), even when the t-SNE perplexity parameter was varied widely. In addition, independently from the t-SNE analysis, Gap statistics [17] from the average linkage hierarchical clustering at different tree cuts resulting in clusters of varying cardinalities displays similar grouping of cell lines. Furthermore, the inflection points in the number of clusters formed at different thresholds demonstrate that the cell lines are organized into distinct clusters based on expression vector similarity (S3 Fig). Next, we asked: to what extent the RPPA and transcriptomic data is concurrent. Although the cell line distances for protein and mRNA data were correlated (r = 0.58), they were surprisingly different for particular pairs of cell lines (Fig 1C). To quantify these differences and rank cell lines with the most characteristic protein or transcript signatures, we calculated the residuals of a linear regression between the protein and mRNA cell line distances. According to this model, we found that the 49 breast cancer cell lines had the largest distances at the protein vs. mRNA levels compared with other sets of cells from other tissues of origin, suggesting that the RPPA measurements better distinguish breast cancer subtypes. Interestingly, hierarchical clustering based on the RPPA data supports three luminal breast cancer subtypes compared with two subtypes identified by transcriptomic data (Fig 1D) here and elsewhere [12]. More broadly, combining data from microarray and RPPA data strengthened the cancer type clustering of cell lines, further suggesting that these measurements of global cellular states are complementary. Overall, clustering of cell lines by transcriptomic and RPPA data is consistent with some cancer types being well-defined and others spanning a wide spectrum of molecular states, while retaining few but important distinguishing differences at both the cancer type and subtype level.

**Fig 2:**
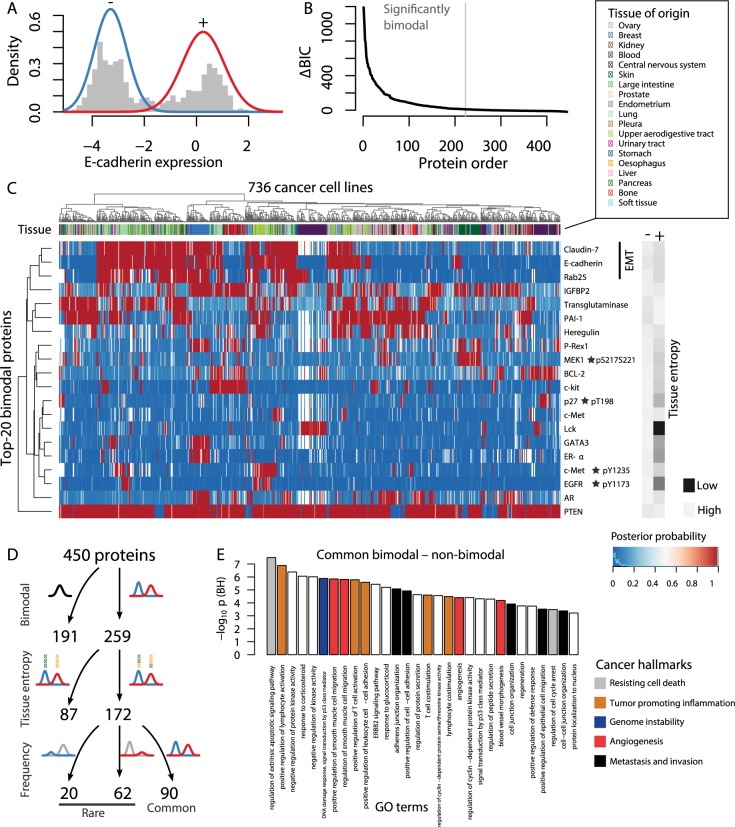
Bimodal protein expression and phosphorylation detected across cancer types associate with known oncogenic processes including EMT. (**A**) Two-component Gaussian mixture model fit to E-cadherin protein expression. The lines indicate the probability density contribution from the low (-) and high (+) expression components. The histogram represents the RPPA measurements for the cell lines. (**B**) By comparing a two- versus one-component fit using the Bayesian Information Criterion (BIC), 260 out of 450 RPPA measurements supported bimodal expression. (**C**) Heat map of the posterior probabilities of each cell line belonging to the low (-, blue) or high (+, red) mixture component for the top-20 most bimodal proteins. The posterior probabilities can be thought of as soft assignments for the cell lines to low or high expression. Shannon entropy of the tissues assigned to low and high expression quantify the tissue diversity giving rise to the bimodal fits. (**D**) Overview of classification approach of proteins in terms of bimodality, tissue diversity (Shannon entropy), and frequency of cell lines assigned to the fitted distributions. (**E**) Significant GO terms for common bimodal proteins that were not found to be significant for non-bimodal proteins (p < 0.05, Benjamini-Hochberg).

To test the hypothesis that EMT governs the molecular states of cell lines across cancer types, we colored the z-scores of E-cadherin expression on the points on the t-SNE maps. For both transcripts and proteins, the cancer cell lines were globally organized by a gradient of E-cadherin expression (Fig 1B, middle). This organization indicates a central role for EMT in characterizing the molecular states of cancer cell lines. Most cancer types associated with common carcinomas had cell lines that spanned this E-cadherin gradient, with lung and breast cancer displaying the largest span. In contrast, cell lines from skin, bone, blood, and kidney were exclusively found in regions with low E-cadherin expression; whereas cell lines from pancreatic and large intestine cancers were found mostly in regions with high E-cadherin expression with only few cell lines expressing E-cadherin at low levels. To ensure the robustness of these findings, we ran independent t-SNE analyses by varying the perplexity parameter, which recapitulated both the E-cadherin gradient and the cancer type-specific clusters (S2 Fig). In comparison, principal component analysis (PCA) yielded less separation of cancer types and a less prominent gradient of E-cadherin expression (S4 Fig). Using cell line annotations from the Catalogue of Somatic Mutations in Cancer (COSMIC), we found no obvious association to whether the cell lines were derived from primary or metastatic tumors (Fig 1B, bottom). This suggests that the arrangement of cell-lines on the t-SNE plots, and thus global expression at the mRNA and protein levels, is dominated by tissue of origin much more than metastatic status. Nonetheless, we propose that collections of pan-cancer cell lines can be used to study aspects of EMT related to E-cadherin expression, which is also clearly bimodal (Fig 2A).

### Bimodal protein expression and phosphorylation indicate oncogenic transitions

Oncogenesis is a multi-step process by which cells acquire cancerous traits, which often mimic physiological cellular processes such as embryogenesis [14]. Such processes are governed by molecular switches that turn on or off coordinated cellular programs. Hence, analyzing the bimodality of protein expression can potentially illuminate cellular states of oncogenesis. To evaluate this idea, we fit a univariate two-component Gaussian mixture model to each RPPA measurement using the expectation-maximization (EM) algorithm. We evaluated bimodality against unimodal distributions using the Bayesian Information Criterion (BIC). Out of the 450 antibody-based RPPA measurements, 260 were bimodal across 736 pan-cancer cell lines (Fig 2B, S1 Table). Among the most bimodal proteins were E-cadherin, Claudin7, and Rab25, all of which have been previously associated with EMT or MET [18]. However, because of the preponderance of tissue-specific signatures among pan-cancer cell lines, bimodal protein expression could more simply be explained by cell type-specific expression. For example, LCK was highly expressed only in a subset of blood cancer cell lines (Fig 2C) concordant with its specific roles in T cell development [19]. To account for residual effects of ancestral cell types, we quantified the tissue diversity of the cell lines assigned to the low- and high expression states by the Shannon entropy of the tissue distributions. We then excluded the lower tertile of the minimum tissue entropy of the low- and high expression states. This approach yielded 172 bimodal proteins and phosphosites (Fig 2B, S1 Table). Out of these, 90 had balanced bimodal distributions including E-cadherin, Claudin7, and Rab25, indicating common pan-cancer oncogenic switches, while 82 were classified as rare transitions (Fig 2D). This filtering and classification is likely prone to false positives due to other confounding factors such as different stages of the circadian clock at time of measurement. Compared to non-bimodal proteins, the proteins associated with the common switches were uniquely enriched for 107 Gene Ontology terms (p < 0.05, after Benjamini-Hochberg correction) many of which can be linked to metastasis and invasion (Fig 2E).

### Coupled bimodality suggests transcript- and protein-based regulatory basis for oncogenic switches

**Fig 3:**
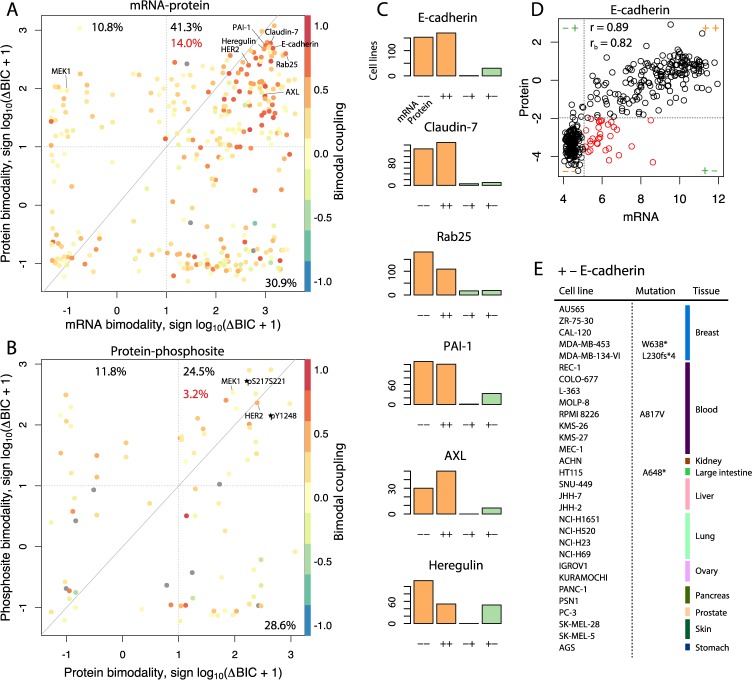
Bimodal coupling between regulatory layers. (**A**) Comparison of mRNA and protein bimodality colored by the coupling (Spearman’s correlation) between posterior probabilities of two-component Gaussian mixture models. The red percentage indicates the fraction of compared genes with coupled (r_b_ > 0.5) bimodalities at the transcript- and protein level. (**B**) Bimodal coupling of phosphosites and protein expression. (**C**) High confidence assignments (p < 0.1) to low or high expression for selection of bimodally coupled mRNA-protein pairs. (**D**) Scatter plot of E-cadherin mRNA and protein expression, indicating in red the 30 cell lines assigned to high mRNA but low protein expression (+–). r is Pearson’s correlation and r_b_ the bimodal coupling coefficient. (**E**) Tissue of origin and *CDH1* (E-cadherin) mutational status of 30 cell lines with high E-cahderin mRNA but low protein expression. Of these cell lines, 3 out of 4 cell lines genotyped for *CDH1* in COSMIC, all had mutations in the coding sequence. fs: frameshift, *: missense.

To identify whether the observed bimodal protein expression across cancer cell lines correlate with transcriptional regulation, we evaluated the bimodality of matching transcripts from CCLE (Fig 3A). We then defined bimodal coupling coefficients between mRNA and protein measurements as the Spearman’s correlation between the posterior probabilities of the mixture model. Overall, 14.0% of proteins measured in MCLP had highly coupled (r_b_ > 0.5) bimodal expression of mRNA and protein. Slightly fewer proteins (10.8%) were uniquely bimodal only at the protein level, including important cancer-related proteins such as MEK1, mTOR, E2F1, TTF1, EIF4G, and JAB1. Hence, these proteins are bimodally expressed due to post-transcriptional regulatory mechanisms such as protein translation and degradation. In addition, we compared the bimodality of proteins and their phosphosites as measured by antibody binding in the RPPA data (Fig 3B). Here, we found weaker bimodal coupling, indicating that phosphosignaling leading to bimodal phosphorylation is mostly independent from basal protein expression. Interestingly, bimodal HER2 phosphorylation at Y1248 was moderately coupled to HER2 protein expression (r_b_ = 0.46), most likely due to autophosphorylation on increased dimerization at higher expression [20]. The bimodal EMT proteins E-cadherin, Claudin7, and Rab25 all had high bimodal mRNA-protein coupling (Fig 3C), confirming that these EMT switches are mostly determined by transcriptional regulation. Nonetheless, 30 cancer cell lines had high expression of the E-cadherin transcript but low protein expression (Fig 3D and 3E), suggesting that E-cadherin could be translationally or post-translationally controlled in some cellular contexts. Among these cell lines, 3 out of 4 *CDH1* genotyped cell lines in COSMIC had either nonsense (MDA-MB-453 and HT115) or frameshift (MDA-MB-134-VI) mutations in *CDH1*, which validate our ability to identify effects on E-cadherin translation. Inactivating mutations in *CDH1* are frequently observed in breast and gastric cancers with cancer type-specific mutational patterns and are associated with loss of cell-cell adhesion and increased cell motility [21]. The nature of the low E-cadherin protein expression in the other 26 cell lines remains unknown, but likely includes inactivating mutations and possibly translational or post-translational regulation.

**Fig 4:**
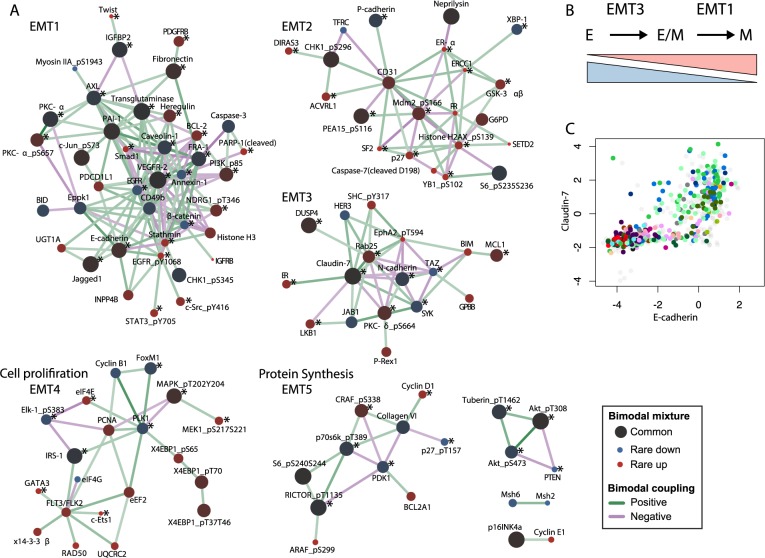
Proteins and phosphosites with coupled bimodality form network communities associated with EMT and intermediate transitions. (**A**) Pan-cancer protein communities detected by Spearman’s correlation of the posterior probabilities of cell lines having low or high expression (|r_b_| > 0.3). Only RPPA measurements associated with bimodal fits with high tissue diversity were included. Network communities were detected by calculating the leading non-negative eigenvector according to Newman’s method. Only edges within identified communities are shown, colored by the magnitude of the bimodal coupling coefficients. The size and color of the nodes represent the fitted mixing parameters from the Gaussian mixture models, quantifying whether underlying switches are common or rare in cancer cell lines. Each community was manually named according to plausible biological mechanisms by conducting a literature search for their protein members. Asterisks (*) indicate proteins with reported mechanisms linked to EMT. (**B**) Proposed interpretation of a two-step transition from the endothelial–E, to the mesenchymal–M states through two identified modules: EMT1 and EMT3. (**C**) Supporting protein expression data showing that Claudin7 and E-cadherin are correlated. Cell lines are colored by the tissue of origin (see Fig 3 for tissue labels).

Transcriptional mechanisms that determine molecular switches are regulated by upstream signaling, such as phosphorylation cascades, which leads to coordinated expression of multiple genes. To detect candidates for such signaling and further characterize the EMT-related states in cancer cell lines, we analyzed the network of bimodal coupling coefficients among bimodal protein and phosphosites associated with high tissue diversity. We first trimmed the protein network by including only significant bimodal coupling coefficients (FDR < 5%, Bonferroni) with |r_b_| > 0.3. This yielded a network of 172 protein nodes connected by 507 edges, from which network communities were defined based on the leading non-negative eigenvector [22]. In total, we detected 8 protein communities that likely reflects shared underlying signaling or cellular events (Fig 4A). One community (EMT1) was clearly linked to EMT, containing E-cadherin, β-catenin, Fibronectin and Twist among several other EMT-related proteins (Fig 4A). E-cadherin was connected to EPPK1, INPP4B, Stathmin, Jagged1, UGT1A, and PDCD1L1, indicating that these might be involved in EMT. The strong bimodal coupling to EPPK1 could help explain why loss of E-cadherin is associated with migratory phenotypes and not just loss of cell-cell adherence; in mice keratinocytes, EPPK1 knockout cells exhibit faster migration and increased wound healing [23]. E-cadherin was also positively coupled to the phosphosite EGFR pY1068, which was in turn positively coupled to SRC pY416 and STAT3 pY705, suggesting a role for phosphorylation of these sites in EMT. Strikingly, all detected communities contained multiple proteins with known mechanisms linking them to EMT but also identified potentially undiscovered components (Fig 4A). The dispersion of these EMT-related proteins among the identified protein communities suggests that they are either part of separate biological processes, or that their involvement in EMT depends on cancer subtypes. Another intriguing possibility is that the multiple protein communities associated with EMT reflect partial cellular states in-between epithelial and mesenchymal phenotypes (Fig 4B). In support of this idea, P-cadherin has previously been suggested as a marker of metastable EMT states [24]. Here we find P-cadherin in the EMT2 community (Fig 4A). In addition, Claudin7 is highly coupled to E-cadherin (r_b_ = 0.70), but found in a separate community (EMT3), along with Rab25 and N-cadherin. Looking closer at this correlation, cell lines had high E-cadherin and low Claudin7 expression but not conversely (Fig 4C). Two other communities are identified, EMT4 and EMT5. The EMT4 cluster contains key cell-cycle transcription-factors such as FoxM1, Cyclin-B1, and Elk1, together with protein kinases that are known to positively regulate their activity, including PLK1, MAPK, and MEK1. Consequently, this cluster indicates changes in cell proliferation regulation. The EMT5 cluster contains a clique made of 3 protein kinases known to regulate the protein translation machinery: RICTOR, P70S6K, and PDK1; and S6 a key protein in the 40S ribosomal subunit. Hence, this cluster likely represents changes in protein translation activity related to overall cell growth. It should also be noted that highly studied proteins and phosphoproteins such pAKT, Cyclin D1, PTEN, and PKC are known to be central to many other pathways, not just to EMT. Hence, labeling all identified clusters as EMT clusters needs to be considered with such general functions in mind. Altogether, it is possible that the protein communities EMT1 and EMT3 may reflect a two-step transition (Fig 4B). In summary, the quintessential EMT marker E-cadherin was found centrally in a large protein and phosphosite network community with clear associations to known EMT markers. For these reasons, we focused subsequent analyses around the expression of E-cadherin, arguing that this approach reflects core aspects of EMT that are invariant across cancer types.

### E-cadherin and bimodally coupled mRNA define genome-wide EMT profile

**Fig 5:**
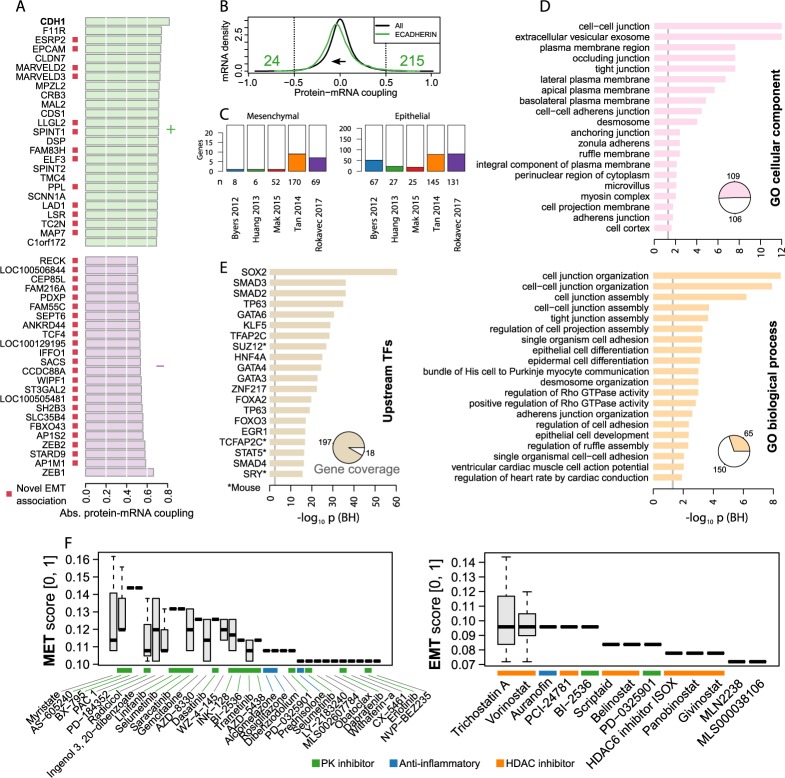
Pan-cancer bimodal coupling between E-cadherin protein expression and genome-wide transcripts defines an EMT signature, predicting EMT- and MET-inducing small-molecules. (**A**) Top-25 transcripts in CCLE with the strongest positive and top 24 negative bimodal coupling coefficients (r_b_) to E-cadherin protein expression. Red squares indicate previous EMT signature genes in non-small cell lung carcinoma published by Byers *et al*. [51]. To define an EMT signature, we considered transcripts with |r_b_| > 0.5, resulting in 215 epithelial and 24 mesenchymal markers. (**B**) Distribution of bimodal coupling coefficients, showing that E-cadherin coupling coefficients are shifted towards negative values compared to all measured proteins. (**C**) Overlap of EMT signature with previously published transcriptomic EMT signatures. The ‘mesenchymal’ bar plot is for the inversely correlated (coupled) genes and the ‘epithelial’ for the positively correlated genes. (**D**-**E**) Gene set enrichment analysis of epithelial part of the EMT signature. The TF enrichment analysis used ChIP-seq data to predict TFs involved in the regulation of the epithelial genes. The pie charts indicate the fraction of the signature genes associated with significantly enriched terms or TFs. (**F**) Small-molecule perturbations predicted to induce EMT and MET based on L1000 cell line data and the L1000CDS^2^ method. The top-50 signatures are shown with results from multiple cell lines or concentrations aggregated by boxplots. PK: protein kinase.

Because E-cadherin is primarily transcriptionally controlled, we next sought to characterize the coordinated transcriptional program associated with E-cadherin down-regulation. First, we defined an EMT signature based on the bimodal coupling (|r_b_| > 0.5) between E-cadherin protein expression and transcriptomic measurements from CCLE, resulting in 239 transcripts—215 positively and 24 negatively coupled (Fig 5A, S2 Table). To our knowledge, these 239 genes include many novel epithelial and mesenchymal markers, while recovering many known EMT markers previously described (Fig 5C), for example, Axl which was reported for non-small cell lung carcinoma [25], or KPNA2 for ovarian carcinoma [26]. The preponderance of positively coupled transcripts suggests that the EMT signature is predominantly characterized by down-regulation of genes governing epithelial traits rather than by gain of mesenchymal traits. Nonetheless, the bimodal coupling coefficients were shifted towards negative values (Fig 5B) and we did find negatively coupled mesenchymal markers such as ZEB1/2 [4].

To further characterize the EMT signature, we performed enrichment analysis on the epithelial markers (Fig 5B). Enrichment analysis [27, 28] for Gene Ontology (GO) cellular components and biological processes clearly demonstrated epithelial phenotypes (Fig 5D). We also found enrichment for localization to the perinuclear region, which is a cytosolic region next to the nuclear envelope with largely unknown composition and biological function. This suggests that the epithelial markers can be used to prioritize spatial cellular regions not widely considered to be affected by EMT such as the perinuclear region, where components of endocytosis aggeregate, although it is well established that endocytosis is central to cell migration. Enrichment for TF binding, using aggregated results from ChIP-seq studies [29], identified SOX2, SMAD2-4, TP63, GATA3-4, and GATA6, which likely act to down-regulate epithelial genes during EMT (Fig 5E). The identified enriched TFs OCT4, SOX2, NANOG, KLF4, and ESRRB are all known to be essential for maintaining pluripotency of human and mouse embryonic stem cells [30]. These TFs bind to super-enhancer regions and through the Mediator complex [31]. Therefore, large parts of the observed transcriptional bimodality could be explained by TF co-operation at super-enhancers resulting in switch-like regulation at numerous genomic loci. At the *CDH1* loci, ENCODE ChIP-seq data supports the involvement of super-enhancers since the loci is marked by high H3K27ac correlated with E-cadherin expression (S5 Fig). Previously, super-enhancers have been proposed to control partial EMT through the putative master regulator TFs ETS2, HNF4A, and JUNB [32], the first two of which we also identified through the TF enrichment analyses. Taken together, pan-cancer bimodality uncovers oncogenic states and regulatory mechanisms of EMT and MET.

### EMT and MET can potentially be induced by small molecules

Gene expression-based, high-throughput screening is a promising approach to identifying small-molecule candidates that can reverse or mimic changes in expression observed in transition to a disease state [33]. To detect small molecules that would maximally push cells toward the EMT or MET expression state, we queried the EMT signature against signatures from ~20,000 small-molecule perturbations of ~50 human cell lines generated by the library of network-based cellular signatures (LINCS) project L1000 dataset [34]. We searched for small molecules that down-regulate epithelial genes and up-regulate mesenchymal genes, resulting in candidate EMT inducers (Fig 5E). Small molecules with the opposite effects were interpreted as MET inducers. Strikingly, most small molecules predicted to induce EMT were HDAC inhibitors, whereas most small molecules predicted to induce MET were kinase inhibitors. The identified HDAC inhibitor Trichostatin A has been shown to induce EMT in prostate cancer cells through modification of H3 near promoters of EMT-related genes [35]. Of the candidate MET inducers, Selumetinib, Trametinib, and PD-0325901 are thought to inhibit MEK, while Saracatinib and Dasatinib to inhibit SRC among other kinome targets. In agreement with these findings, a prior high-content chemical screen aimed at identifying inhibitors of EMT has predominantly identified other similar kinase inhibitors based on cell growth and migration assays [36]. Hence, in summary, caution should be placed in utilizing HDAC inhibitors as therapeutics due to their putative potential to enhance EMT as predicted by chemogenomics screening.

### Causal protein and phosphorylation models identify drivers of cancer signaling and progression

**Fig 6:**
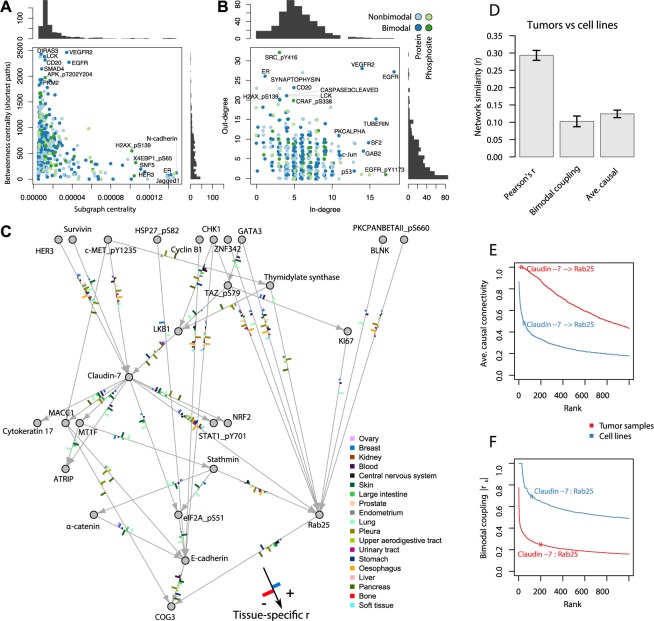
Bayesian networks of proteins and phosphosites inferred from pan-cancer cell lines identify drivers of EMT and correlate to tumor networks. All Bayesian network structures were inferred by a Fast Greedy Search algorithm. (**A**) Network centrality statistics of the directed causal graph over all measured proteins pertaining to the influence of proteins on cancer signaling. (**B**) In and out degree distributions of proteins and phosphosites from the inferred network. (**C**) Causal neighborhood (1st neighbors) of EMT markers E-cadherin, Rab25, and Claudin7. Tissue-specific correlations in support for each edge are shown as bars along the edges. The layout was determined using the hierarchical Sugiyama algorithm with all edges oriented downwards. (**D**) Network comparisons between cell line and tumor data. (**E**) Distribution of average connectivity in bootstrapped Bayesian networks. (**F**) Distribution of networks of bimodal coupling coefficients.

The bimodal coupling model we implemented to analyze EMT is essentially correlative and hence not causal. However, establishing causal interactions based on RPPA data is challenging without time-series or direct perturbation data such as gene knock-downs or knock-outs [37]. With sufficient sample size and coverage of diverse cell lines, it is in principle possible to identify causal, regulatory interactions between measured signaling components. Despite not satisfying the observation that cell signaling regulatory networks contain feedback loops [38], learning Bayesian network learning algorithms can be applied to construct causal models of cellular regulatory networks, including cell signaling networks, from observational data [39–41]. To infer causal relationships among proteins and phosphosites measured by RPPA, we used a Fast Greedy Search algorithm to estimate a Bayesian network over all 450 RPPA measurements (Fig 6). Based on the resulting directed acyclic graph, we calculated betweenness centrality, subgraph centrality, in-degree, and out-degree for each analyte. Bimodal phosphosites overall had higher betweenness centrality (p = 0.036, t-test). By considering measures of network influence, several proteins and phosphosites were identified as promising candidate drivers (Fig 6A and 6B). In particular, SRC pY416 had the highest out-degree. This phosphosite is known to be highly predictive of patient survival [42].

Furthermore, we analyzed the network neighborhood of E-cadherin, Claudin7, and Rab25, which spanned the proposed two-step transition: EMT3 followed by EMT1 (Fig 6C). Using a hierarchical network layout algorithm, E-cadherin was found downstream of Claudin7 and Rab25 concurrent with EMT3 preceding EMT1 in cancer cell lines. In addition, several proteins and phosphosites upstream of the EMT markers were plausible oncogenic drivers for specific cancer types, supported by prior reports. For example, MACC1 is associated with pancreatic EMT and metastasis [43], which the analysis found for both pancreatic and pleural cancer cell lines, while suggesting the opposite effect in lung, endometrium, and upper digestive tract cancers. Lastly, we also found LKB1, CHK1, and Stathmin upstream of EMT markers. The inactivation of LKB1, which is frequently mutated in lung adenocarcinomas, induces EMT in lung cancer cells through activation of ZEB1 [44], whereas CHK1 mediates DNA damage response as part of EMT by stabilizing ZEB1 [45]. Inhibiting the microtubule destabilizer Stathmin impedes EMT by increased microtubule formation [46]. In conclusion, inferred Bayesian protein networks based on pan-cancer cell lines can potentially identify key drivers of EMT.

To validate the causal models, we carried out bootstrapping for 200 iterations and considered the average network for both cell line and tumor data (Supporting Information, S3 Table, Fig 6E). Although the reproducibility of particular edges in the ensemble of Bayesian networks was relatively low for the cell line data, the connection from Claudin7 to Rab25 was present in 48% of the networks, and in all the networks inferred from tumor samples. In contrast, the statistical reproducibility of the tumor networks was higher, most likely due to the larger sample size (n = 3,161). Overall, the average Bayesian networks were significantly correlated between cell line and tumor data (Fig 6D). The bimodal coupling coefficients, however, were lower in the tumor data, indicating that bimodal expression is less pervasive in tumors compared to cell lines. This result might indicate that tumors, more-so than cell lines, contain mixtures of cell types that are in multiple cellular states. Overall, the learning Bayesian Network strategy employed here is exploratory and needs further evaluation, parameter tuning and validation.

## Discussion

Cellular transitions from epithelial to mesenchymal phenotypes share common characteristics such as down-regulation of E-cadherin in a variety of tissues and cancer contexts [47]. In this study, we demonstrate that in pan-cancer cell lines, bimodal coupling of transcript, protein, and phosphosite expression reveal epithelial and mesenchymal states. However, many known EMT markers are dispersed across bimodally coupled network modules, suggesting that they are involved in distinct regulatory programs. Different modules likely correspond to intermediate cellular states of an EMT spectrum, whereby transcriptional down-regulation of E-cadherin, and other genes, represents a decisive loss of epithelial traits. In agreement, we find that E-cadherin expression is primarily transcriptionally controlled, possibly with context-dependent control at the translational or post-translational level. By anchoring the investigation around the transcriptional program associated with E-cadherin down-regulation, we identified 239 bimodal EMT markers, many of which have not previously been associated with EMT.

The observation that EMT markers are particularly bimodal suggests that cell lines are unequivocally either epithelial or mesenchymal in cell culture. It follows that the EMT decision for cells is determined by the growth medium and the genetics of the cancer cells, rather than by stochastic processes leading to heterogeneous mixtures of cells. Therefore, the identified bimodal switches likely reflect deterministic rather than stochastic architecture. However, in bulk experiments of cell lines, rare but important populations of cells such as mesenchymal stem cells could be neglected. The lack of obvious association between metastasis and E-cadherin expression raises some questions. Possibly, the *in vitro* conditions, lack of cues from tumor microenvironments, and cell culture passages might mask the original metastatic events from which the cell line is derived. More broadly, cell culture conditions may fail to model crucial aspects of how EMT occurs in complex tissue environments. Yet, the identified deterministic mechanisms may be valid only under the right conditions.

We find that cancer cell lines down-regulate epithelial and up-regulate mesenchymal genes when treated with HDAC inhibitors. This observation warrants caution for the use of HDAC inhibitors as cancer and other therapies. If HDAC inhibitors induce EMT in cancer cells, this could explain the disappointing outcomes in clinical trials of HDAC monotherapies for solid tumors [48]. Several protein kinase inhibitors were predicted to revert cancer cell lines to a more epithelial state, but most of these kinase inhibitors are not currently in clinical use. Therefore, these kinase inhibitors may be effective as metastatic repressors and could be under-investigated due to the contemporary focus on targeted drug treatments rather than broad functional effects. Furthermore, the mechanisms of action for the small-molecules may inform us about EMT or MET drivers. For example, the identification that the two SRC inhibitors Dasatinib and Saracatinib are potentially MET inducers, the co-clustering of SRC pY416 with Claudin7, and its large out-degree in the Bayesian network, all corroborate evidence to the importance of SRC activity for regulating EMT during cancer progression. In addition, the identification of kinase inhibitors rather than other classes of small-molecules suggests that phospho-signaling in general is particularly important for driving MET.

Lastly, we show that causal models of protein expression and phosphorylation in cancer cell lines identify known and putative drivers of EMT. Due to the promising preliminary results from the causal models that we constructed, identifying molecular drivers of EMT, despite the lack of statistical power to robustly detect individual causal interactions, it is clear that measuring more cell lines, under more conditions, would substantially increase the sensitivity and in turn quality of such models. Also, if a sufficient number of pan-cancer cell lines could be profiled by mass spectrometry proteomics, the developed bimodal methodology could be reapplied to confirm and discover novel associations between proteins and post-translational modifications that drive oncogenic state transitions.

## Methods

### Matching RPPA and CCLE cell line data

The RPPA data for 736 cancer cell lines were generated at the MD Anderson Cancer Center. The selection criteria of the 474 measured proteins were based on the aim to cover known cancer-related signaling pathways. We excluded antibodies with missing values across cell lines by requiring that each RPPA measurement is present in at least 40 cell lines. This resulted in a dataset with 450 antibody-based measurements. The CCLE mRNA data and cell line annotations of 1,037 cancer cell lines were retrieved from the CCLE portal at: https://portals.broadinstitute.org/ccle. We used the gene-centric RMA-normalized data.

### Cell line distances and clustering

For all methods relying on geometric distances, Euclidean distances were computed considering only pairwise complete features. Sparse RPPA measurements were excluded, requiring that each protein is measured in at least 100 cell lines, which resulted in the inclusion of 263 protein measurements. To reduce the dimensionality of the RPPA and mRNA data, we used t-SNE implemented in the R package ‘tsne’ with perplexity value of 30 and at 5,000 iterations, and all other arguments at their default values [16]. Only cell lines with available RPPA and mRNA data were included. For the combined RPPA and CCLE mRNA embedding, the distance matrices for each data set were weighted by the sum of all distances. In this way, each data type contributed equally to the combined analysis. To more rigorously assess the number of clusters supported by the RPPA and CCLE data sets, we calculated the Gap statistic [17] from the average linkage hierarchical clustering at tree cuts resulting in clusters of varying cardinalities. PCA was performed using the R package ‘pcaMethods’ from data that were centered and scaled to unit variance, while imputing missing values with the ‘svdImpute’ method. Furthermore, we analyzed patterns in the classification and misclassification of the tissue of origin for the RPPA and mRNA data using 3-nearest-neighbor classification according to a leave-one-out cross-validation scheme. Linear regression was carried out between the RPPA and CCLE mRNA distance matrices with the RPPA distances considered the target variable. The residuals of the regression thus quantify the deviation from expected distance for RPPA data for each pairwise cell line distance. To compare the clustering of breast cancer cell lines, we computed tanglegrams using the dendextend R package. The tanglegram method uses a random search to rotate tree nodes minimizing the overlap of lines drawn between leaves of two trees.

### Statistical models of bimodal expression

We fit univariate two-component Gaussian distributions using the expectation-maximization (EM) algorithm implemented in the ‘mixtools’ R package with default parameters. To compare the fitted distribution to unimodal Gaussian distributions, we calculated the difference between the Bayesian Information Criterion (BIC). The data were determined to be bimodal if the BIC difference was larger than 2. Based on the fitted Gaussian mixture model, we calculated, using Bayes’ theorem, the posterior probabilities of measurements being generated from the high expression component. Note that the probability of belonging to the low component is 1-p. To estimate the tissue diversity of each bimodal fit, we first calculated the frequencies of tissues assigned to the low (p < 0.5) and the high (p > = 0.5) component. We then calculated the Shannon entropy of the tissue distributions associated with the low- and the high components. The bimodal RPPA measurements were classified into groups of low, medium, and high tissue diversity by the tertiles of the minimum tissue entropy associated with low- and high expression. The bimodal expression was considered common if the fitted mixture coefficients were above 1/4 and rare if below. Based on the posterior probabilities of the bimodal fits associated with high tissue diversity, we calculated a network of bimodal coupling coefficients defined as Spearman’s correlations between the posterior probabilities. To detect robust communities in this network, we set a cutoff of |r_b_| > 0.3 and calculated the leading non-negative eigenvectors using the igraph R package. The network was visualized in Cytoscape with node size proportional to the mixing parameter of the two-component Gaussian fit and with edge coloring based on the coupling coefficients.

### EMT signature enrichment and queries

The coupling coefficients between the E-cadherin RPPA measurements and matched CCLE transcript data were used to define an EMT signature (r_b_ > 0.5). Enrichment analysis was performed with Enrichr [28]. L1000CDS2 was used to query small molecules as potential inducers or reversers of EMT [34]. We summarized the EMT and MET small-molecule predictions by reporting the top-50 small molecules identified using boxplots to aggregate small molecules with multiple experimental conditions such as cell lines, dosage, or timing.

### Causal modeling

The causal models of the RPPA measurements across cancer cell lines were inferred using the Fast Greedy Search algorithm [49] implemented by the BD2K Center for Causal Discovery [50]. We used the rcausal R package version 0.99.5 to run the Java implementation with penalty discount 4 and depth 3. To visualize causal neighborhoods, we computed graph cuts and rendered the subnetwork in R using a Sugiyama layout of the directed acyclic graph. The tissue-specific correlations were layered on top of the edges as histograms. To estimate the robustness of the resulting causal network, we ran the algorithm several times in a bootstrap scheme (M = 200) by sampling with replacement.

## Supporting information

S1 Fig Misclassification of cancer types classified by nearest neighbor classification. (EPS)

S2 Fig Independent t-SNE analyses while varying the perplexity parameter. (EPS)

S3 Fig Gap statistics from the average linkage hierarchical clustering at different tree cuts. Arrows point to infection points where the cell lines are organized into clusters. Such inflection points are not expected for random distances between cell lines.
(EPS)

S4 Fig Principal component analysis (PCA) of cell lines colored by gradient of E-cadherin expression. (EPS)

S5 Fig Genome browser tracks around the *CDH1* gene. (EPS)

S1 Table Bimodal RPPA proteins across the 736 pan-cancer cell lines. (XLSX)

S2 Table EMT signature of 239 transcripts with 215 positively and 24 negatively coupled transcripts. (XLSX)

S3 Table Causal network edge list for the network learned using the Bayesian network modeling. (CSV)

## References

[1] Potenta S, Zeisberg E, Kalluri R. The role of endothelial-to-mesenchymal transition in cancer progression. British journal of cancer. 2008;99(9):1375–9. doi: 10.1038/sj.bjc.6604662 18797460 10.1038/sj.bjc.6604662 PMC2579683

[2] Nieto MA, Huang RY-J, Jackson RA, Thiery JP. EMT: 2016. Cell. 2016;166(1):21–45. doi: 10.1016/j.cell.2016.06.028 27368099 10.1016/j.cell.2016.06.028

[3] Tan TZ, Miow QH, Miki Y, Noda T, Mori S, Huang RYJ, et al Epithelial‐mesenchymal transition spectrum quantification and its efficacy in deciphering survival and drug responses of cancer patients. EMBO molecular medicine. 2014;6(10):1279–93. doi: 10.15252/emmm.201404208 25214461 10.15252/emmm.201404208 PMC4287932

[4] Tam WL, Weinberg RA. The epigenetics of epithelial-mesenchymal plasticity in cancer. Nature medicine. 2013;19(11):1438–49. doi: 10.1038/nm.3336 24202396 10.1038/nm.3336 PMC4190672

[5] Kalluri R, Weinberg RA. The basics of epithelial-mesenchymal transition. The Journal of clinical investigation. 2009;119(6):1420–8. doi: 10.1172/JCI39104 19487818 10.1172/JCI39104 PMC2689101

[6] Batlle E, Sancho E, Francí C, Domínguez D, Monfar M, Baulida J, et al The transcription factor snail is a repressor of E-cadherin gene expression in epithelial tumour cells. Nature cell biology. 2000;2(2):84–9. doi: 10.1038/35000034 10655587 10.1038/35000034

[7] Peinado H, Ballestar E, Esteller M, Cano A. Snail mediates E-cadherin repression by the recruitment of the Sin3A/histone deacetylase 1 (HDAC1)/HDAC2 complex. Molecular and cellular biology. 2004;24(1):306–19. doi: 10.1128/MCB.24.1.306-319.2004 14673164 10.1128/MCB.24.1.306-319.2004 PMC303344

[8] Puisieux A, Brabletz T, Caramel J. Oncogenic roles of EMT-inducing transcription factors. Nature cell biology. 2014;16(6):488–94. doi: 10.1038/ncb2976 24875735 10.1038/ncb2976

[9] Tibes R, Qiu Y, Lu Y, Hennessy B, Andreeff M, Mills GB, et al Reverse phase protein array: validation of a novel proteomic technology and utility for analysis of primary leukemia specimens and hematopoietic stem cells. Molecular cancer therapeutics. 2006;5(10):2512–21. doi: 10.1158/1535-7163.MCT-06-0334 17041095 10.1158/1535-7163.MCT-06-0334

[10] Li J, Zhao W, Akbani R, Liu W, Ju Z, Ling S, et al Characterization of Human Cancer Cell Lines by Reverse-phase Protein Arrays. Cancer Cell. 2017;31(2):225–39. doi: 10.1016/j.ccell.2017.01.005 28196595 10.1016/j.ccell.2017.01.005 PMC5501076

[11] Barretina J, Caponigro G, Stransky N, Venkatesan K, Margolin AA, Kim S, et al The Cancer Cell Line Encyclopedia enables predictive modelling of anticancer drug sensitivity. Nature. 2012;483(7391):603–7. doi: 10.1038/nature11003 22460905 10.1038/nature11003 PMC3320027

[12] Weinstein JN, Collisson EA, Mills GB, Shaw KRM, Ozenberger BA, Ellrott K, et al The cancer genome atlas pan-cancer analysis project. Nature genetics. 2013;45(10):1113–20. doi: 10.1038/ng.2764 24071849 10.1038/ng.2764 PMC3919969

[13] Li J, Lu Y, Akbani R, Ju Z, Roebuck PL, Liu W, et al TCPA: a resource for cancer functional proteomics data. Nature methods. 2013;10(11):1046–7. doi: 10.1038/nmeth.2650 Epub 2013 Sep 15. 24037243 10.1038/nmeth.2650 PMC4076789

[14] Rokavec M, Kaller M, Horst D, Hermeking H. Pan-cancer EMT-signature identifies RBM47 down-regulation during colorectal cancer progression. Scientific Reports. 2017 7 5;7(1):4687 doi: 10.1038/s41598-017-04234-2 28680090 10.1038/s41598-017-04234-2 PMC5498532

[15] Ramaswamy S, Tamayo P, Rifkin R, Mukherjee S, Yeang C-H, Angelo M, et al Multiclass cancer diagnosis using tumor gene expression signatures. Proceedings of the National Academy of Sciences. 2001 12 18;98(26):15149–54. Epub 2001 Dec 11. 11742071 10.1073/pnas.211566398 PMC64998

[16] Lvd Maaten, Hinton G. Visualizing data using t-SNE. Journal of Machine Learning Research. 2008;9(Nov):2579–605.

[17] Tibshirani R, Walther G, Hastie T. Estimating the number of clusters in a data set via the gap statistic. Journal of the Royal Statistical Society: Series B (Statistical Methodology). 2001;63(2):411–23.

[18] Bhat A, Pope J, Smith J, Ahmad R, Chen X, Washington M, et al Claudin-7 expression induces mesenchymal to epithelial transformation (MET) to inhibit colon tumorigenesis. Oncogene. 2015;34(35):4570–80. doi: 10.1038/onc.2014.385 25500541 10.1038/onc.2014.385 PMC4804637

[19] Palacios EH, Weiss A. Function of the Src-family kinases, Lck and Fyn, in T-cell development and activation. Oncogene. 2004;23(48):7990 doi: 10.1038/sj.onc.1208074 15489916 10.1038/sj.onc.1208074

[20] Brennan PJ, Kumogai T, Berezov A, Murali R, Greene MI. HER2/neu: mechanisms of dimerization/oligomerization. Oncogene. 2000;19(53):6093 doi: 10.1038/sj.onc.1203967 11156522 10.1038/sj.onc.1203967

[21] Berx G, Becker KF, Höfler H, Van Roy F. Mutations of the human E‐cadherin (CDH1) gene. Human mutation. 1998;12(4):226–37. doi: 10.1002/(SICI)1098-1004(1998)12:4<226::AID-HUMU2>3.0.CO;2-D 9744472 10.1002/(SICI)1098-1004(1998)12:4<226::AID-HUMU2>3.0.CO;2-D

[22] Newman ME. Modularity and community structure in networks. Proceedings of the national academy of sciences. 2006 6 6;103(23):8577–82. Epub 2006 May 24. 16723398 10.1073/pnas.0601602103 PMC1482622

[23] Goto M, Sumiyoshi H, Sakai T, Fässler R, Ohashi S, Adachi E, et al Elimination of epiplakin by gene targeting results in acceleration of keratinocyte migration in mice. Molecular and cellular biology. 2006;26(2):548–58. doi: 10.1128/MCB.26.2.548-558.2006 16382146 10.1128/MCB.26.2.548-558.2006 PMC1346887

[24] Ribeiro AS, Paredes J. P-cadherin linking breast cancer stem cells and invasion: a promising marker to identify an “intermediate/metastable” EMT state. Frontiers in oncology. 2015;4:371 doi: 10.3389/fonc.2014.00371 25601904 10.3389/fonc.2014.00371 PMC4283504

[25] Byers LA, Diao L, Wang J, Saintigny P, Girard L, Peyton M, et al An epithelial–mesenchymal transition gene signature predicts resistance to EGFR and PI3K inhibitors and identifies Axl as a therapeutic target for overcoming EGFR inhibitor resistance. Clinical cancer research. 2013;19(1):279–90. doi: 10.1158/1078-0432.CCR-12-1558 23091115 10.1158/1078-0432.CCR-12-1558 PMC3567921

[26] Huang L, Wang H, Li J, Wang J, Zhou Y, Luo R, et al KPNA2 promotes cell proliferation and tumorigenicity in epithelial ovarian carcinoma through upregulation of c-Myc and downregulation of FOXO3a. Cell death & disease. 2013 8 1;4:e745 doi: 10.1038/cddis.2013.256 23907459 10.1038/cddis.2013.256 PMC3763430

[27] Chen EY, Tan CM, Kou Y, Duan Q, Wang Z, Meirelles GV, et al Enrichr: interactive and collaborative HTML5 gene list enrichment analysis tool. BMC bioinformatics. 2013 4 15;14:128 doi: 10.1186/1471-2105-14-128 23586463 10.1186/1471-2105-14-128 PMC3637064

[28] Kuleshov MV, Jones MR, Rouillard AD, Fernandez NF, Duan Q, Wang Z, et al Enrichr: a comprehensive gene set enrichment analysis web server 2016 update. Nucleic acids research. 2016 7 8;44(W1):W90–7. doi: 10.1093/nar/gkw377 Epub 2016 May 3. 27141961 10.1093/nar/gkw377 PMC4987924

[29] Lachmann A, Xu H, Krishnan J, Berger SI, Mazloom AR, Ma'ayan A. ChEA: transcription factor regulation inferred from integrating genome-wide ChIP-X experiments. Bioinformatics. 2010;26(19):2438–44. doi: 10.1093/bioinformatics/btq466 20709693 10.1093/bioinformatics/btq466 PMC2944209

[30] MacArthur BD, Ma'ayan A, Lemischka IR. Systems biology of stem cell fate and cellular reprogramming. Nature Reviews Molecular Cell Biology. 2009;10(10):672–81. doi: 10.1038/nrm2766 19738627 10.1038/nrm2766 PMC2928569

[31] Whyte WA, Orlando DA, Hnisz D, Abraham BJ, Lin CY, Kagey MH, et al Master transcription factors and mediator establish super-enhancers at key cell identity genes. Cell. 2013;153(2):307–19. doi: 10.1016/j.cell.2013.03.035 23582322 10.1016/j.cell.2013.03.035 PMC3653129

[32] Chang H, Liu Y, Xue M, Liu H, Du S, Zhang L, et al Synergistic action of master transcription factors controls epithelial-to-mesenchymal transition. Nucleic acids research. 2016;44(6):2514–27. doi: 10.1093/nar/gkw126 26926107 10.1093/nar/gkw126 PMC4824118

[33] Stegmaier K, Ross KN, Colavito SA, O'Malley S, Stockwell BR, Golub TR. Gene expression-based high-throughput screening (GE-HTS) and application to leukemia differentiation. Nature genetics. 2004;36(3):257 doi: 10.1038/ng1305 14770183 10.1038/ng1305

[34] Duan Q, Reid SP, Clark NR, Wang Z, Fernandez NF, Rouillard AD, et al L1000CDS2: LINCS L1000 Characteristic Direction Signatures Search Engine. npj Systems Biology and Applications. 2016;2:16015 doi: 10.1038/npjsba.2016.15 28413689 10.1038/npjsba.2016.15 PMC5389891

[35] Kong D, Ahmad A, Bao B, Li Y, Banerjee S, Sarkar FH. Histone deacetylase inhibitors induce epithelial-to-mesenchymal transition in prostate cancer cells. PLoS One. 2012;7(9):e45045 doi: 10.1371/journal.pone.0045045 23024790 10.1371/journal.pone.0045045 PMC3443231

[36] Chua K-N, Sim W-J, Racine V, Lee S-Y, Goh BC, Thiery JP. A cell-based small molecule screening method for identifying inhibitors of epithelial-mesenchymal transition in carcinoma. PloS one. 2012;7(3):e33183 doi: 10.1371/journal.pone.0033183 22432005 10.1371/journal.pone.0033183 PMC3303807

[37] Hill SM, Heiser LM, Cokelaer T, Unger M, Nesser NK, Carlin DE, et al Inferring causal molecular networks: empirical assessment through a community-based effort. Nature methods. 2016;13(4):310 doi: 10.1038/nmeth.3773 26901648 10.1038/nmeth.3773 PMC4854847

[38] Bhalla US, Iyengar R. Emergent properties of networks of biological signaling pathways. Science. 1999;283(5400):381–7. 9888852 10.1126/science.283.5400.381

[39] Sachs K, Perez O, Pe'er D, Lauffenburger DA, Nolan GP. Causal protein-signaling networks derived from multiparameter single-cell data. Science. 2005;308(5721):523–9. doi: 10.1126/science.1105809 15845847 10.1126/science.1105809

[40] Koller D, Friedman N. Probabilistic graphical models: principles and techniques: MIT press; 2009.

[41] Cooper GF, Herskovits E. A Bayesian method for the induction of probabilistic networks from data. Machine learning. 1992;9(4):309–47.

[42] Akbani R, Ng PKS, Werner HM, Shahmoradgoli M, Zhang F, Ju Z, et al A pan-cancer proteomic perspective on The Cancer Genome Atlas. Nature communications. 2014 5 29;5:3887 doi: 10.1038/ncomms4887 24871328 10.1038/ncomms4887 PMC4109726

[43] Wang G, Kang M-X, Lu W-J, Chen Y, Zhang B, Wu Y-L. MACC1: A potential molecule associated with pancreatic cancer metastasis and chemoresistance. Oncology letters. 2012;4(4):783–91. doi: 10.3892/ol.2012.784 23205101 10.3892/ol.2012.784 PMC3506703

[44] Roy BC, Kohno T, Iwakawa R, Moriguchi T, Kiyono T, Morishita K, et al Involvement of LKB1 in epithelial–mesenchymal transition (EMT) of human lung cancer cells. Lung Cancer. 2010;70(2):136–45. doi: 10.1016/j.lungcan.2010.02.004 20207041 10.1016/j.lungcan.2010.02.004

[45] Zhang P, Wei Y, Wang L, Debeb BG, Yuan Y, Zhang J, et al ATM-mediated stabilization of ZEB1 promotes DNA damage response and radioresistance through CHK1. Nature cell biology. 2014;16(9):864–75. doi: 10.1038/ncb3013 25086746 10.1038/ncb3013 PMC4150825

[46] Li N, Jiang P, Du W, Wu Z, Li C, Qiao M, et al Siva1 suppresses epithelial–mesenchymal transition and metastasis of tumor cells by inhibiting stathmin and stabilizing microtubules. Proceedings of the National Academy of Sciences. 2011 8 2;108(31):12851–6. doi: 10.1073/pnas.1017372108 Epub 2011 Jul 18. 21768358 10.1073/pnas.1017372108 PMC3150944

[47] Lamouille S, Xu J, Derynck R. Molecular mechanisms of epithelial–mesenchymal transition. Nature reviews Molecular cell biology. 2014;15(3):178 doi: 10.1038/nrm3758 24556840 10.1038/nrm3758 PMC4240281

[48] West AC, Johnstone RW. New and emerging HDAC inhibitors for cancer treatment. The Journal of clinical investigation. 2014;124(1):30 doi: 10.1172/JCI69738 24382387 10.1172/JCI69738 PMC3871231

[49] Chickering DM, Meek C, editors. Finding optimal bayesian networks Proceedings of the Eighteenth conference on Uncertainty in artificial intelligence; 2002: Morgan Kaufmann Publishers Inc.

[50] Cooper GF, Bahar I, Becich MJ, Benos PV, Berg J, Espino JU, et al The center for causal discovery of biomedical knowledge from big data. Journal of the American Medical Informatics Association. 2015 11;22(6):1132–6. doi: 10.1093/jamia/ocv059 Epub 2015 Jul 2. 26138794 10.1093/jamia/ocv059 PMC5009908

[51] Byers LA, Wang J, Nilsson MB, Fujimoto J, Saintigny P, Yordy J, et al Proteomic profiling identifies dysregulated pathways in small cell lung cancer and novel therapeutic targets including PARP1. Cancer discovery. 2012 9;2(9):798–811. doi: 10.1158/2159-8290.CD-12-0112 Epub 2012 Sep 6. 22961666 10.1158/2159-8290.CD-12-0112 PMC3567922

